# NKCC-1 mediated Cl^−^ uptake in immature CA3 pyramidal neurons is sufficient to compensate phasic GABAergic inputs

**DOI:** 10.1038/s41598-020-75382-1

**Published:** 2020-10-27

**Authors:** Sergey N. Kolbaev, Namrata Mohapatra, Rongqing Chen, Aniello Lombardi, Jochen F. Staiger, Heiko J. Luhmann, Peter Jedlicka, Werner Kilb

**Affiliations:** 1 Institute of Physiology, University Medical Center Mainz, Johannes Gutenberg University, Duesbergweg 6, 55128 Mainz, Germany; 2 Institute of Clinical Neuroanatomy, Neuroscience Center, Goethe-University, Theodor-Stern-Kai 7, 60590 Frankfurt, Germany; 3 Institute of Neuroanatomy, Universitätsmedizin Göttingen, Georg-August-Universität Göttingen, Kreuzbergring 36, 37075 Göttingen, Germany; 4 ICAR3R-Interdisciplinary Centre for 3Rs in Animal Research, Faculty of Medicine, Justus-Liebig-University, Rudolf-Buchheim-Str. 6, 35392 Giessen, Germany; 5 Present Address: Research Center of Neurology, Volokolamskoyeshosse, 80, Moscow, Russia 125367; 6 Present Address: Department of Neurobiology, School of Basic Medical Sciences, Southern Medical University, Guangzhou, 510515 China

**Keywords:** Computational biology and bioinformatics, Developmental biology, Neuroscience

## Abstract

Activation of GABA_A_ receptors causes in immature neurons a functionally relevant decrease in the intracellular Cl^−^ concentration ([Cl^−^]_i_), a process termed ionic plasticity. Amount and duration of ionic plasticity depends on kinetic properties of [Cl^−^]_i_ homeostasis. In order to characterize the capacity of Cl^−^ accumulation and to quantify the effect of persistent GABAergic activity on [Cl^−^]_i_, we performed gramicidin-perforated patch-clamp recordings from CA3 pyramidal neurons of immature (postnatal day 4–7) rat hippocampal slices. These experiments revealed that inhibition of NKCC1 decreased [Cl^−^]_i_ toward passive distribution with a time constant of 381 s. In contrast, active Cl^−^ accumulation occurred with a time constant of 155 s, corresponding to a rate of 15.4 µM/s. Inhibition of phasic GABAergic activity had no significant effect on steady state [Cl^−^]_i_. Inhibition of tonic GABAergic currents induced a significant [Cl^−^]_i_ increase by 1.6 mM, while activation of tonic extrasynaptic GABA_A_ receptors with THIP significantly reduced [Cl^−^]_i._. Simulations of neuronal [Cl^−^]_i_ homeostasis supported the observation, that basal levels of synaptic GABAergic activation do not affect [Cl^−^]_i_. In summary, these results indicate that active Cl^−^-uptake in immature hippocampal neurons is sufficient to maintain stable [Cl^−^]_i_ at basal levels of phasic and to some extent also to compensate tonic GABAergic activity.

## Introduction

GABA (γ-amino butyric acid) is a major inhibitory neurotransmitter in the central nervous system of mammals^1^ and is involved in the regulation of excitation, control of motor output or sensory integration, generation of oscillatory activity, neuronal assembly formation, and neuronal plasticity^2^. The responses to GABA are mediated via metabotropic GABA_B_ receptors and ionotropic GABA_A_ and GABA_C_ receptors. GABA_A/C_ receptors represent ligand-gated anion-channels with a high permeability for Cl^−^ ions, while HCO_3_^−^ ions contribute only partially to the ionic currents^1^. During early neuronal development GABA_A_ receptor-mediated responses are depolarizing, due to a high [Cl^−^]_i_^3^. This high [Cl^−^]_i_ is maintained by the activity of the isoform 1 of the Na^+^-dependent K^+^, Cl^−^ Cotransporter (NKCC1), which mediates an uptake of Cl^−^^4–8^. The depolarizing GABAergic responses in the immature hippocampus have been associated with the generation of spontaneous oscillatory activity transients that are essential for brain development^9,10^, but also to the higher incidence and pharmacological refractoriness of epileptic seizures in the immature CNS^11–14^.

A variety of studies have demonstrated that the Cl^−^-fluxes through GABA_A_ receptors influence [Cl^−^]_i_ on a shorter time scale and thus temporarily affect the amplitude of subsequent GABAergic responses^15–19^. This process is termed ionic plasticity^5,20^. Such activity-dependent [Cl^−^]_i_ transients have been implicated in a variety of pathophysiological processes^21–23^, but they also underlie physiological functions, e.g. in the developing spinal cord where transient activity-dependent collapses of the Cl^−^ gradient generate slow oscillatory activity^7,24^. The size of activity-dependent Cl^−^ transients is on the first instance determined by the relation between Cl^−^ influx and the capacity of Cl^−^ extrusion systems^5,25^. Several computational studies revealed that, in addition to a variety of morphological and/or electrophysiological properties^26–28^, the capacity of the transmembrane Cl^−^-transport is the main factor determining the spatiotemporal dynamics as well as the final amount of activity-dependent alterations in [Cl^−^]_i_^23,26,27,29–31^. Therefore, a quantification of the transport capacity of Cl^−^-extrusion or -uptake is necessary for a better understanding of GABAergic and glycinergic function during early development.

However, to our knowledge a detailed investigation of the kinetic properties of Cl^−^-transport has only been published for Cajal-Retzius cells^4^ and immature spinal-cord motoneurons^7^ as well as for mature cultured hippocampal^23^ and thalamic neurons^32^. Whereas in mature hippocampal neurons the [Cl^−^]_i_ relaxation upon a massive [Cl^−^]_i_ increase occurs rather fast within ~ 30s^23^, in immature neurons a rather inefficient Cl^−^-transport was observed^4,7^. In these neurons the recovery after [Cl^−^]_i_ depletion requires ~ 10–20 min^4,7^. Accordingly, it has been shown for both, Cajal-Retzius cells and immature motoneurons, that physiological levels of neuronal activity can cause functionally relevant changes in [Cl^−^]_i_^19,33^. Another striking example for activity-dependent changes in [Cl^−^]_i_ has been described in the immature hippocampus, where the massive GABAergic synaptic drive during giant depolarizing potentials, a network phenomenon essential for the development of hippocampal connectivity^34^, causes massive alterations in [Cl^−^]_i_^17^. However, while several studies have already reported that the high [Cl^−^]_i_ of immature hippocampal neurons is maintained by NKCC1^14,35,36^, to our knowledge no information about the kinetic properties of Cl^−^-transport in immature hippocampal neurons is available yet.

Therefore, we performed gramicidin-perforated patch-clamp recordings from visually identified CA3 pyramidal neurons of immature (postnatal day [P] 4–7) rat hippocampus to quantify the active and passive transport rates for Cl^−^ in immature hippocampal neurons. In addition, we analyzed whether baseline levels of synaptic and extrasynaptic GABAergic activity can influence [Cl^−^]_i_. Since the massive [Cl^−^]_i_ transients caused by GDPs would impair the analysis of the kinetic properties of [Cl^−^]_i_ homeostasis, we performed these experiments in coronal hippocampal slices, as in these slices only a fraction of connectivity is maintained^37^ and therefore they fail to generate GDPs. Our experiments revealed that the rate of transmembrane Cl^−^-transport is rather low in immature CA3 neurons. Nevertheless, these transport rates are sufficient to maintain a stable [Cl^−^]_i_ during baseline synaptic GABAergic activity, while the physiological levels of tonic GABAergic currents provoke a slight shift in [Cl^−^]_i_.

## Results

### Steady-state distribution of [Cl^−^]_i_ in immature CA3 pyramidal cells

**Figure 1:**
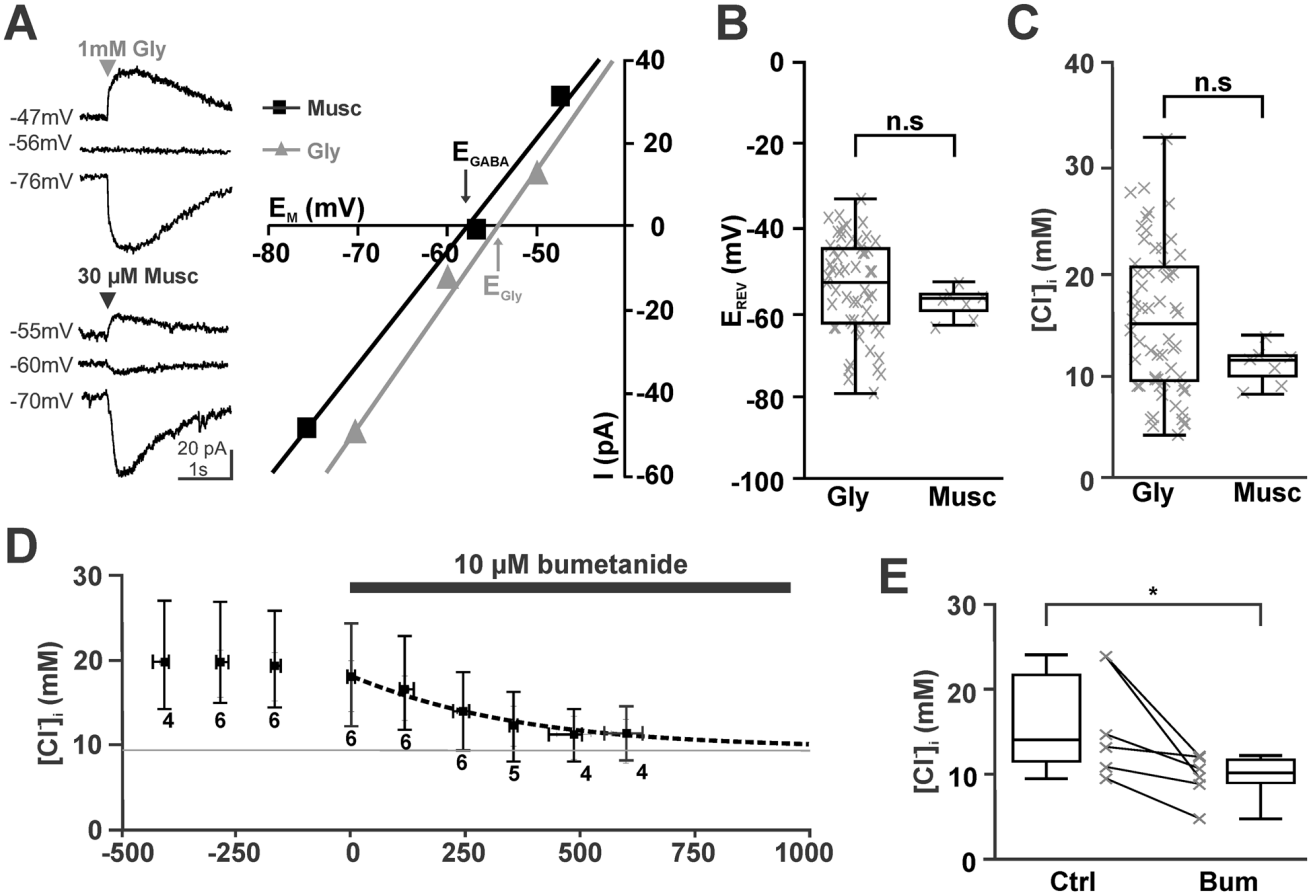
Inhibition of NKCC1 with bumetanide leads to a slow [Cl^−^]_i_ decrease. (**A**) Determination of reversal potentials for GABAergic (black) and glycinergic (gray) currents measured under gramicidin-perforated conditions. The left traces represent typical current traces upon focal application of 1 mM glycine (upper traces) and 30 µM muscimol (lower traces). Only 3 agonist applications at 3 different holding potentials were performed to minimize the effects of agonist–evoked Cl^−^ currents on [Cl^−^]_i_. (**B**) Box plot diagrams illustrating that glycinergic and GABAergic reversal potentials are comparable. (C) The [Cl^−^]_i_ calculated from the reversal potentials revealed comparable values for both agonists. (**D**) Bath application of 10 µM bumetanide induced an exponential decrease of [Cl^−^]_i_ (dashed line) towards the passive distribution (gray line). Data points represent median ± interquartile range, number of experiments are indicated below the error bars. E: Statistical analysis demonstrating the reliable decrease of [Cl^−^]_i_ in all experiments.

In this study we recorded in total from 121 CA3 pyramidal cells under gramicidin-perforated patch-clamp conditions. We estimated the reversal potential of GABAergic (E_GABA_) and glycinergic (E_Gly_) currents from short (2–10 ms) puffs of 30 µM muscimol or 0.2–1 mM glycine applied focally to the soma of the pyramidal cells (Fig. 1A). The use of glycine pulses was necessary for the determination of [Cl^−^]_i_ in part of the following experiments, as in these experiments gabazine or picrotoxin were used to eliminate phasic and tonic GABAergic currents. At a holding potential of − 70 mV these cells showed an E_GABA_ of − 57 [-55.6, -60.0] mV (n = 7) or an E_Gly_ of − 52 [-44.6, -62.7] mV (n = 58, Fig. 1B). Both values were not significantly different (*p* = 0.315, Mann–Whitney). Using published values for the HCO_3_^−^ permeability of GABA (0.18) or glycine (0.11) receptors^38^ and the estimated extra- and intracellular HCO_3_^−^ concentrations, these values correspond to a [Cl^−^]_i_ of 11.5 [9.8, 11.8] mM (n = 7) and 14.8 [9.4, 20.3] mM (n = 58), respectively (Fig. 1C). These [Cl^−^]_i_ values were also not significantly different (*p* = 0.117, Mann–Whitney).

In order to determine the kinetics of passive Cl^−^-efflux and to confirm that this high [Cl^−^]_i_ was maintained by the activity of the NKCC1^14,35^, we first analyzed the effect of the NKCC1 inhibitor bumetanide on [Cl^−^]_i_. Bath application of 10 µM bumetanide resulted in a significant (*p* = 0.028, Wilcoxon) decline of [Cl^−^]_i_ from 14.5 [11.5, 21.6] mM (n = 6) to 10.2 [9.0, 11.8] mM (n = 6) within ~ 10 min (Fig. 1D,E). This decline in the [Cl^−^]_i_ could be fitted by a monoexponential function using a τ of 381 s (Fig. 1D). From this function we estimated a maximal passive Cl^−^ efflux of 15.5 µM/s at a [Cl^−^]_i_ of ~ 15 mM. The [Cl^−^]_i_ of 10.2 [9.0, 11.8] mM (n = 6) obtained in the presence of bumetanide is in the range of the passive Cl^−^ distribution of 9.1 mM. In summary, this result confirms that NKCC1 substantially contribute to the active Cl^−^ accumulation and is counteracting a passive Cl^−^ efflux^5,8^.

### Estimation of the capacity of NKCC1 mediated Cl^−^ uptake

**Figure 2:**
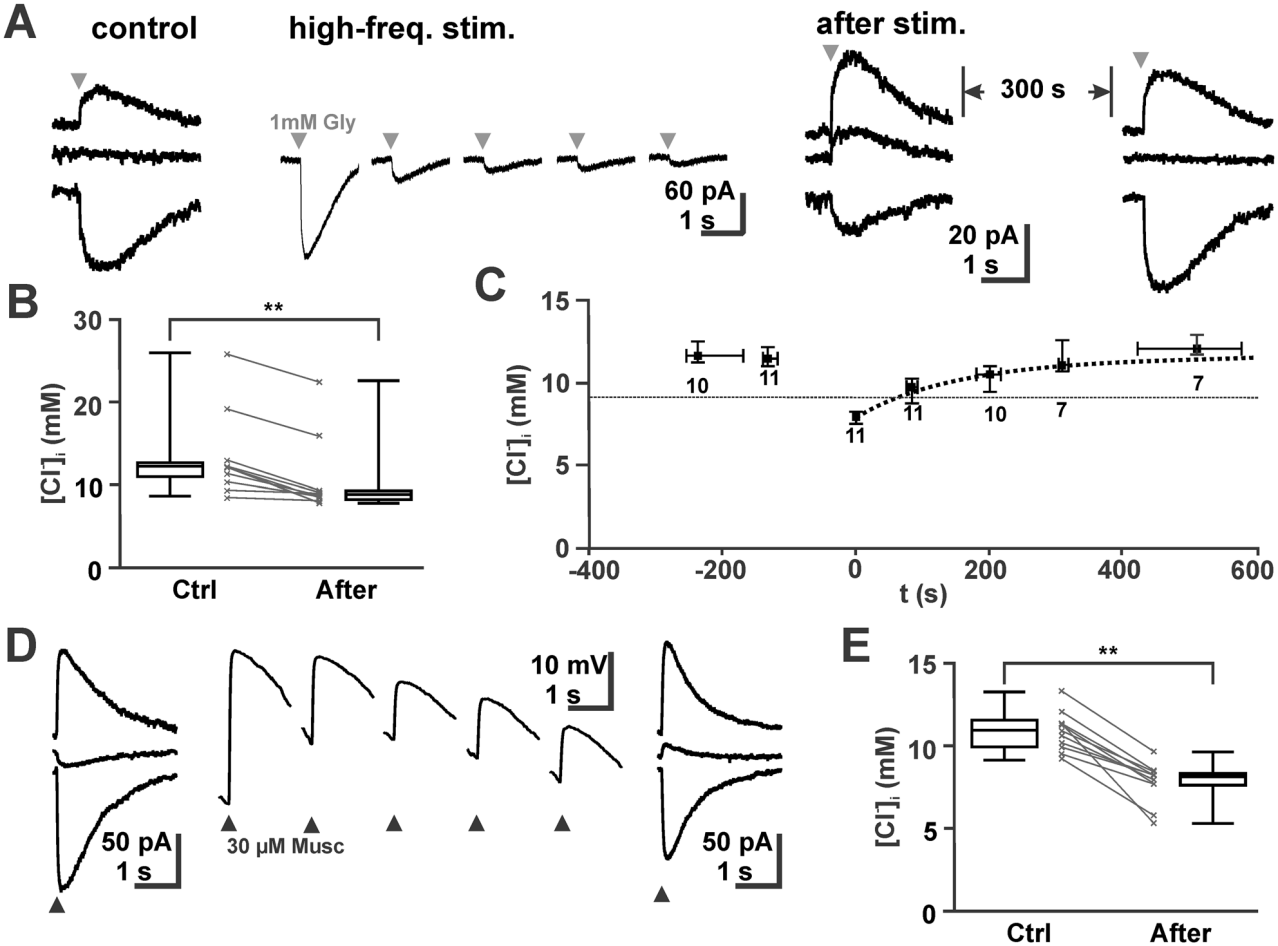
Slow recovery of [Cl^−^]_i_ after depletion. (**A**) Typical current traces upon application of 1 mM glycine at holding potentials of − 43, − 53 and − 73 mV. The thin traces represent representative current traces during the unloading protocol. Note the obvious shift in E_REV_ after the unloading protocol, which reversed after 300 s. (**B**) Statistical analysis demonstrating the reliable decrease of [Cl^−^]_i_ in all experiments upon repetitive stimulation. (**C**) Analysis of [Cl^−^]_i_ before and after the stimulation protocol revealed that [Cl^−^]_i_ recovered with an exponential time course (dashed line) towards the baseline [Cl^−^]_i_. Data points represent median ± interquartiles, number of experiments are indicated below the error bars. (**D**) Typical current traces upon application of 30 µM muscimol. Note the obvious shift in E_REV_ after repetitive muscimol application under current-clamp conditions. (**E**) Analysis of [Cl^−^]_i_ before and after repetitive muscimol application revealed that [Cl^−^]_i_ was significantly reduced after massive GABAergic stimulation.

Since the kinetics of [Cl^−^] transport is one major factor influencing ionic plasticity^17,26–28,39,40^, we next determined the kinetic properties of Cl^−^ uptake after an artificial [Cl^−^]_i_ reduction. To quantify the kinetics of the [Cl^−^]_i_ reuptake we decreased [Cl^−^]_i_ by 25 pulses of either 1–3 mM glycine or 30 µM muscimol applied with a frequency of 0.5 Hz to voltage-clamped neurons (Fig. 2A). This procedure significantly (*p* = 0.002, Wilcoxon) reduced the [Cl^−^]_i_ by 3.2 [2.6, 3.8] mM (n = 12) from 11.4 [10.3, 11.8] mM to 7.9 [7.7, 8.4] mM (Fig. 2B). As neither the amount of the [Cl^−^]_i_ decrease (*p* = 0.808, Mann–Whitney) nor the time constants of [Cl^−^]_i_ recovery (*p* = 0.291, Mann–Whitney) were significantly different between glycine- and muscimol-application experiments, the data was pooled. The subsequent recovery of [Cl^−^]_i_ was monitored by determining E_REV_ with a small number of test pulses given at intervals of ~ 100 s, to avoid substantial Cl^−^ fluxes by these test pulses^4^. These experiments showed that [Cl^−^]_i_ returned to the resting values within ~ 10 min (Fig. 2C). This increase in [Cl^−^]_i_ could be described with a monoexponential function using a time constant τ of 155 s. At a [Cl^−^]_i_ of 9.1 mM, which represents a passive distribution at − 70 mV holding potential and thus eliminates passive fluxes, the active Cl^−^ uptake rate amounted to 15.4 µM/s. In summary, these results indicate that NKCC1-mediated Cl^−^ uptake is sufficient to maintain [Cl^−^]_i_ at the observed values, but that this transport process is rather slow in immature CA3 pyramidal neurons.

### Effect of spontaneous phasic GABAergic activity

Given the low capacity of NKCC1-mediated Cl^−^-uptake, we next investigate whether GABAergic activity can override [Cl^−^]_i_ homeostatic processes and thus influence the steady-state [Cl^−^]_i_ levels. In accordance with previous publications that demonstrated substantial [Cl^−^]_i_ changes upon frequent activation of GABA_A_ receptors^15,16^, we observed that the repetitive application of 25 muscimol (30 µM) pulses with a frequency of 0.5 Hz led to a significant (*p* = 0.003, Wilcoxon) decrease in [Cl^−^]_i_ by 3.1 [2.2, 3.7] mM (n = 11) (Fig. 2D,E).

**Figure 3:**
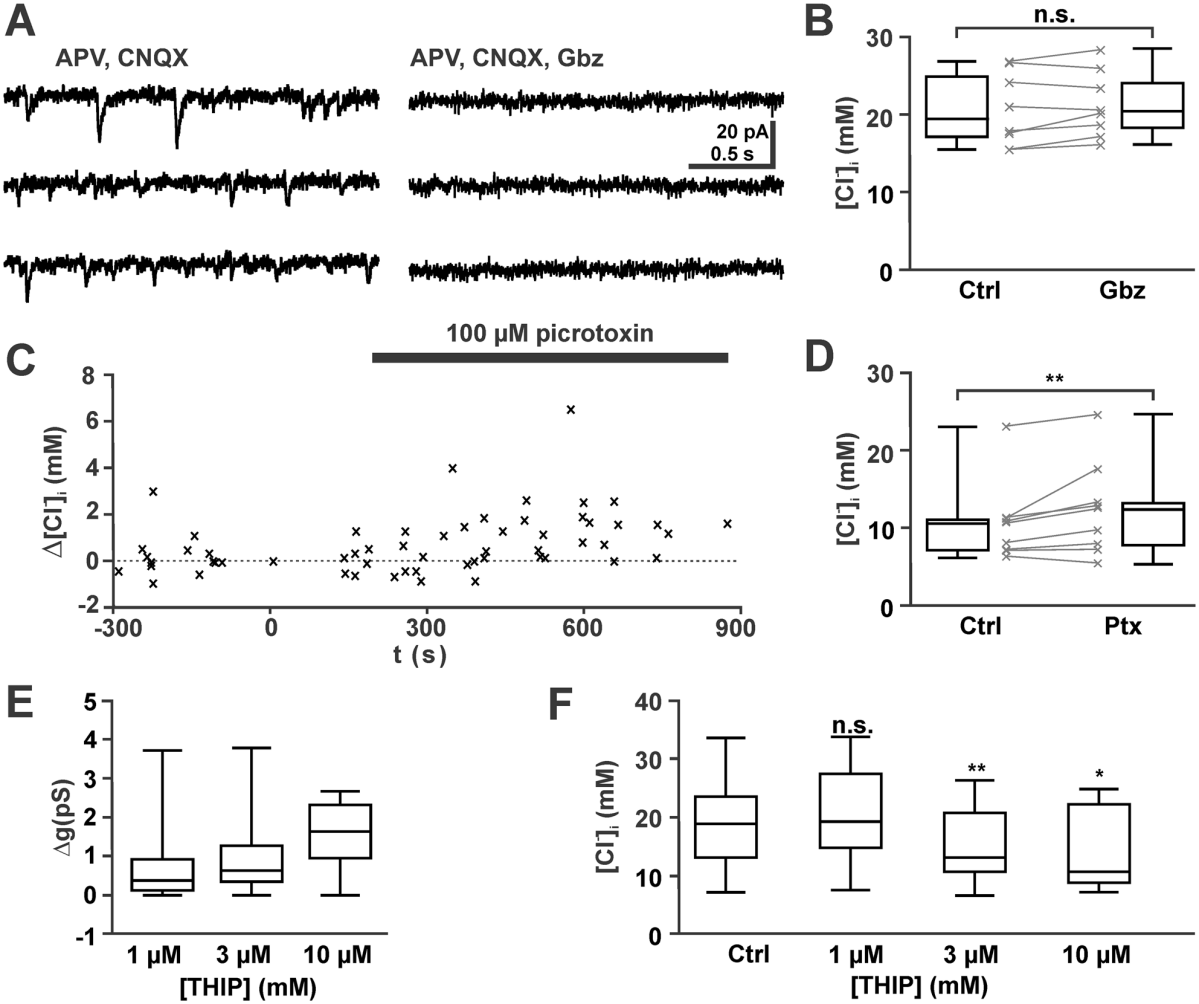
No effect of phasic (synaptic) and mild effect of tonic GABAergic activity on [Cl^−^]_i_. (**A**) Typical current traces illustrating that pharmacologically isolated GABAergic PSCs were completely suppressed by 1 µM gabazine (Gbz). (**B**) Statistical analysis illustrating that a complete suppression of GABAergic PSCs has no significant effect on [Cl^−^]_i._. (**C**) Shift in [Cl^−^]_i_ upon bath application of 100 µM picrotoxin (Ptx) under voltage-clamp conditions. Ptx inhibits both tonic (extrasynaptic) as well as phasic (synaptic) GABAergic currents. Each symbol represents an individual data point from n = 9 experiments. The [Cl^−^]_i_ was related to the [Cl^−^]_i_ in the last measurement before Ptx application. Note the tendency toward an increased [Cl^−^]_i_ in the presence of Ptx. (**D**) Statistical analyses of these experiments revealed an increased [Cl^−^]_i_ after the onset of Ptx application. (**E**) Bath application of THIP dose-dependently increased the membrane conductance. THIP enhances tonic (extrasynaptic) GABAergic conductance. F: Statistical analysis illustrating that THIP induced a dose-dependent decrease in [Cl^−^]_i_.

Since this result indicates that GABAergic activity has the potential to contribute to [Cl^−^]_i_ homeostasis, we next investigated whether the observed levels of spontaneous synaptic (phasic) GABAergic activity or the tonic GABAergic conductance influence the resting [Cl^−^]_i_ of pyramidal cells. For this purpose, GABAergic current are isolated by bath application of the glutamatergic antagonists CNQX (30 µM) and APV (20 µM). In line with previous studies reporting a moderate frequency of GABA_A_ mediated synaptic events in the immature hippocampus^13,41,42^, the frequency of pharmacologically isolated GABAergic PSCs in the present study was 2.03 [1.28, 2.03] Hz (n = 6 cells with 1531 events), with a median amplitude of 8.5 [7.3, 14.8] pA (corresponding to a peak conductance of 122 [105.4, 213.6] pS). These GABAergic PSCs were completely suppressed in the presence of 1 µM gabazine (Fig. 3A), which at this concentration selectively blocks synaptic GABA_A_ receptors^13^. To unravel whether an inhibition of synaptic GABAergic activity influences [Cl^−^]_i_, we determined [Cl^−^]_i_ before and after synaptic GABAergic activity was inhibited for 5—16 min under current clamp conditions. These experiments revealed that after a complete blockade of spontaneous GABAergic inputs, [Cl^−^]_i_ was non-significantly (*p* = 0.161, Wilcoxon test) altered by 0.7 [-0.5, 1.6] mM (n = 8) from 18.3 [15.8, 23.8] mM to 19.2 [17.1, 23.0] mM (Fig. 3B). Note that the tendency (*p* = 0.07, Mann–Whitney U-test) to higher basal [Cl^−^]_i_ in these neurons, as compared to all recordings, led to a higher driving force for Cl^−^ ions and would thus result in even higher activity-dependent [Cl^−^]_i_ changes. We conclude from these results that the capacity of NKCC1-mediated Cl^−^ uptake in immature CA3 pyramidal neurons is sufficient to cope with the Cl^−^-influx caused by spontaneous GABAergic synaptic inputs.

Tonic currents mediated by extrasynaptic GABA receptors contribute substantially to passive [Cl^−^]_i_ fluxes in immature neurons ^4^, as such tonic currents mediate a larger charge transfer^43^. Since in the immature hippocampus extrasynaptic receptors substantially contribute to the excitability^9,13,41^, we also investigated whether tonic GABAergic currents influence [Cl^−^]_i_. For this purpose, we blocked tonic and phasic GABAergic currents with picrotoxin^13^. Bath application of 100 µM picrotoxin unravelled a tonic GABAergic conductance of 0.9 [0.49, 1.01] pS (n = 9). The continuous application of 100 µM PTX for 5–12 min led under voltage-clamp conditions to a slight, but significant (*p* = 0.008, Wilcoxon test) increase in [Cl^−^]_i_ by 1.6 [0.7, 1.9] mM (n = 9) from 10.8 [7.3, 11.3] mM to 12.7 [8.1, 13.5] mM (Fig. 3C,D).

In line with this observation, enhancement of tonic conductance using THIP led to a small decrease in [Cl^−^]_i_. To avoid the induction of epileptiform activity by THIP^13^, these experiments are performed in the continuous presence of the glutamatergic antagonists CNQX (30 µM) and APV (20 µM). Bath application of 1 µM THIP activated a median membrane conductance of 0.28 [0.03, 0.81] pS (n = 14), which increased to 0.37 [0.09, 1.01] pS (n = 13) and 0.67 [0.02, 1.35] pS (n = 12) in the presences of 3 µM and 10 µM THIP, respectively (Fig. 3E). These enhanced tonic currents led to a significant (*p* = 0.033, Mann–Whitney U-tests) decrease in [Cl^−^]_i_ (Fig. 3F). Upon a constant THIP application for 4 – 12 min [Cl^−^]_i_ decreased in the presence of 3 µM THIP by 3.0 [1.8, 6.9] mM (n = 13, *p* = 0.009, Wilcoxon test) and in 10 µM THIP by 5.0 [0.5, 10.3] mM (n = 12, *p* = 0.015, Wilcoxon test). In the presence of 1 µM THIP no significant (*p* = 0.638, Wilcoxon test) [Cl^−^]_i_ alteration (0.09 [-3.4, 4.4] mM, n = 14) was observed. In summary, these results suggest that basal levels of tonic GABAergic activity can induce Cl^−^ fluxes that influence mildly the resting [Cl^−^]_i_.

### Estimation of activity dependent [Cl^−^]_i_ alterations using compartmental modeling

**Figure 4:**
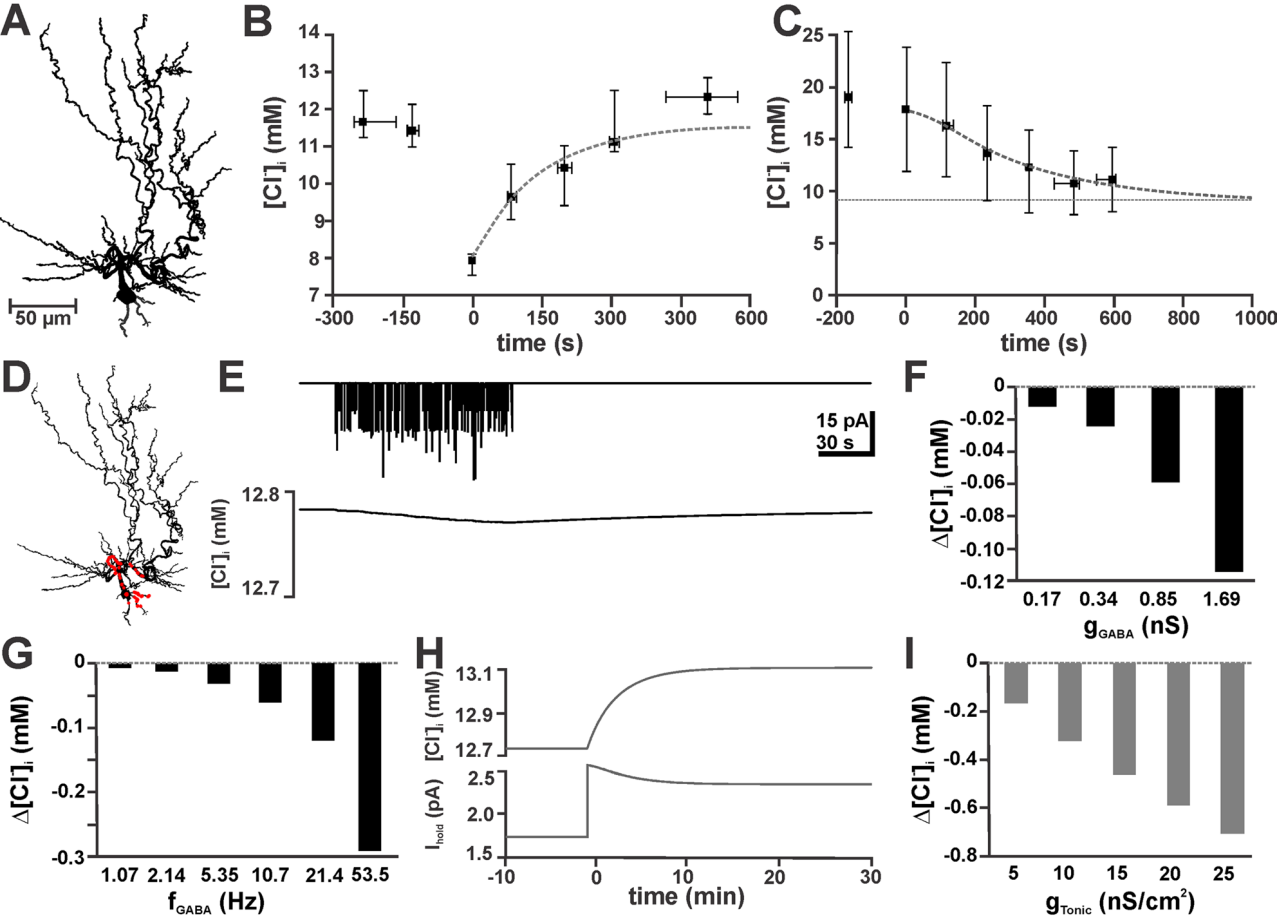
A biophysically realistic compartmental model of active Cl^−^uptake and diffusion confirmed limited alterations in [Cl^−^]_i_ due to phasic and tonic GABAergic currents. (**A**) Morphology of the reconstructed CA3 pyramidal cell used for numerical simulations. (**B**) Fit of the experimentally observed [Cl^−^]_i_ relaxation after Cl^−^ depletion with adequate parameter settings (τ = 78.5 s; [Cl^−^]_i_^0^ = 13.3 mM) for a simulated active Cl^−^ accumulation process (dashed line). (C) Simulation of the experimentally observed [Cl^−^]_i_ decline after elimination of active Cl^−^ accumulation (black symbols) by implementation of a passive tonic Cl^−^ conductance at a density of 8.75 nS/cm^2^ (dashed line) in a simulated neuron lacking active Cl^−^-uptake. (**D**) Representation of the reconstructed neuron in the NEURON model. The locations of GABAergic synapses are indicated by red dots. (**E**) Holding current (upper panel) and [Cl^−^]_i_ (lower panel) upon repetitive synaptic stimulation (g_GABA_ 0.169 nS at 2.14 Hz for 100 s). (**F**) [Cl^−^]_i_ changes induced by 100 s of random synaptic activity with different g_GABA_. (**G**) [Cl^−^]_i_ changes induced by 100 s of random synaptic activity with a g_GABA_ of 0.169 nS at different frequencies. Note that, in line with experiments, physiological values (0.169 nS at 2.14 Hz) induced only marginal [Cl^−^]_i_ changes. (**H**) [Cl^−^]_i_ and holding-current (I_hold_) upon elimination of the basal tonic GABAergic conductance of 8.75 nS/cm^2^ at t = 0. (**I**) [Cl^−^]_i_ changes induced by the addition of a tonic GABAergic component to the basal tonic current.

Finally, we used a data-driven biophysical model of Cl^−^ dynamics to estimate whether Cl^−^ fluxes due to synaptic (phasic) and extrasynaptic (tonic) activation of GABA_A_ channels, transmembrane Cl^−^ transport and Cl^−^ diffusion are able to account for the observed stability of [Cl^−^]_i_. To set the geometry of this computational model we employed the 3D-reconstructed morphology of a young CA3 pyramidal cell (Fig. 4A). The diffusion of Cl^−^ inside the dendritic tree was simulated using deterministic compartmental diffusion modeling implemented in NEURON^40,44^ (see Methods). To simulate tonic GABAergic currents, we added a tonic conductance of 8.75 nS/cm^2^, which allowed the model to replicate the passive Cl^−^ fluxes observed in immature CA3 neurons (Fig. 4B). Next we implemented an active Cl^−^ accumulation process with a τ_Cl_ of 78.5 s and a target [Cl^−^]_i_ ([Cl^−^]_i_^0^) of 13.3 mM, which allowed the modeled neurons to replicate the experimentally determined kinetics of [Cl^−^]_i_ relaxation and the steady-state [Cl^−^]_i_ (Fig. 4C).

To simulate phasic GABAergic activity, we modeled stochastic activation of 107 GABA_A_ synapses located randomly in the soma and perisomatic dendrites^45^ (Fig. 4D). The initial values for the conductance of GABA_A_ synapses (g_GABA_ = 169 pS) and the frequency of GABAergic synaptic currents (2.14 Hz) were based on the mean values of the experimental data. In accordance with the patch-clamp observation, addition of physiological levels of GABAergic synaptic inputs had only a marginal effect on [Cl^−^]_i_. After 100 s of continuous GABAergic activity at 2.14 Hz [Cl^−^]_i_ decreased by only 0.012 mM (Fig. 4E,F). Augmenting g_GABA_ enhanced the synaptically evoked [Cl^−^]_i_ decline (Fig. 4F), which however remained small (0.114 mM) even if g_GABA_ was increased to 1.69 nS. Increasing the frequency of GABAergic synaptic inputs from 2.14 to 5.35 Hz had only a marginal effect of the [Cl^−^]_i_ change (0.031 mM, Fig. 4G), but at frequencies of ~ 20 and ~ 50 Hz [Cl^−^]_i_ changes by 0.12 mM and 0.29 mM, respectively, were induced (Fig. 4G). In summary, these results indicate that basal levels of synaptic activity had no major effect in [Cl^−^]_i_.

To analyze the influence of tonic GABAergic currents, we omitted the tonic GABAergic conductance of 8.75 nS/cm^2^, which changed the holding current (I_hold_) by 0.8 pA and led to a [Cl^−^]_i_ increase by 0.32 mM (Fig. 4H). Enhancing tonic GABAergic currents by adding multiples of 5 nS/cm^2^ to the basal tonic conductance induced a dose-dependent decrease in [Cl^−^]_i_ that, however, remained below 0.8 mM (Fig. 4I). These additional tonic conductances between 5 and 25 nS/cm^2^ induced a linear decrease in the inward current between − 0.3 and − 1.28 pA, respectively.

In summary, these simulations with a realistic computational model of Cl^−^ dynamics support the observation, that NKCC1-mediated Cl^−^ transport is sufficient to maintain [Cl^−^]_i_ at basal levels of phasic GABAergic activity, while tonic currents have a mild effect on steady-state [Cl^−^]_i_.

## Discussion

To comprehend the physiological role of depolarizing GABAergic responses in the immature hippocampus under conditions that dynamically challenge [Cl^−^]_i_ homeostasis, information about the kinetic properties of [Cl^−^]_i_ homeostasis is required^39^. The main findings of the present study can be summarized as follows: (i) NKCC1 is the main Cl^−^ transporter in immature CA3 pyramidal cells and mediates an active Cl^−^-accumulation with a rate of 15.4 µM/s. (ii) Physiological levels of tonic GABAergic activation induce a slight decrease in [Cl^−^]_i_ by 1.6 mM. (iii) In contrast, spontaneous phasic GABAergic PSCs do not significantly affect [Cl^−^]_i_. We conclude from these results, that the [Cl^−^]_i_ of immature CA3 pyramidal neurons represents a steady-state equilibrium between active NKCC1-mediated Cl^−^-accumulation and passive Cl^−^-efflux, mainly via tonic GABAergic currents. The capacity of the NKCC1-mediated Cl^−^ uptake in CA3 pyramidal cells is, however, sufficient to maintain a high [Cl^−^]_i_ under basal levels of GABAergic activity.

In the present study we used relative HCO_3_^−^ permeability ratios of 0.18 for GABA_A_ and of 0.11 for glycine receptors, which were determined by Bormann et al. in cultured spinal cord neurons^38^. While published permeability ratios for GABA_A_ (0.44) and glycine receptors (0.40) in hippocampal neurons are available^46^, using these values resulted in unrealistically low [Cl^−^]_i_ values (ranging from 0.4 to 30 mM). Therefore we prefer to use the values provided by Bormann et al.^38^. Importantly, the qualitative results of our study will not be altered, if the higher relative HCO_3_^−^ permeability ratios are used.

The first outcome of this study is that inhibition of NKCC1 with 10 µM bumetanide induced a decline in [Cl^−^]_i_ towards the passive distribution, which supports the fact that this transporter constitutes the main Cl^−^ uptake mechanism in immature CA3 pyramidal cells^14,35,47^. Bumetanide has been reported to also inhibit other targets, like KCC2 or aquaporins^48,49^, however, at slightly higher effective doses of 55 µM and 100 µM, respectively. An inhibition of these transporters will have no effect on the time constant of the [Cl^−^]_i_ decay upon bumetanide application, but our experiments could not exclude a minor contribution of the [Cl^−^]_i_ extruder KCC2 to steady-state [Cl^−^]_i_ levels. The [Cl^−^]_i_ decline observed in this experiment upon pharmacological inhibition of NKCC1 represents basal Cl^−^ fluxes, which can be mediated via different types of voltage-, volume-, or ligand-gated Cl^−^ channels^50^. Next, we determined the capacity of the NKCC1-mediated Cl^−^-uptake system by quantifying the rate of Cl^−^-accumulation after [Cl^−^]_i_ depletion. Because the [Cl^−^]_i_ recovery depends only on the properties of the uptake systems and should be independent of the agonists used for the induction of the [Cl^−^]_i_ depletion and since the time constants of [Cl^−^]_i_ recovery are not significantly different between glycine- and muscimol-application, data of muscimol- and glycine application experiments were pooled. In these experiments we determined an uptake rate of 15.4 µM/s. This value was comparable to the [Cl^−^]_i_ uptake rate determined previously in immature cortical neurons^4^. In more mature neurons inhibition of transmembrane Cl^−^ transport with furosemide induced a positive shift in E_GABA_, indicating that in slightly older neurons the activity of KCC2 is required to establish a low [Cl^−^]_i_^51^.

The maximal rate for passive Cl^−^ efflux during the [Cl^−^]_i_ decline amounted to ~ 15.5 µM/s and is thus in the range of the Cl^−^-uptake. However, while the effective rate of Cl^−^-uptake was determined at 9.1 mM, which is the passive [Cl^−^]_i_-distribution and thus omits any passive Cl^−^-fluxes, the rate of Cl^−^-efflux was determined at an arbitrary [Cl^−^]_i_ value of 15 mM. Please note, that both fluxes depend on the [Cl^−^]_i_-gradient across the membrane, with the passive fluxes disappearing at 9.1 mM, while the NKCC1-mediated uptake has a considerably lesser dependency of the [Cl^−^]_i_ gradient^5^. Thus the combination of these oppositely directed Cl-fluxes resulted in a steady-state equilibrium^39^, which is maintained at a [Cl^−^]_i_ substantially higher than the passive distribution and thus supports depolarizing GABAergic responses. On the other hand, one should keep in mind that the present study only revealed the properties of the [Cl^−^]_i_ homeostasis in the soma. Variation in the functional expression of active Cl^−^-transport and passive Cl^−^-fluxes may result in different capacities of [Cl^−^]_i_ homeostasis in distinct compartments.

Further experiments of the present study revealed, that inhibition of phasic, synaptic GABAergic inputs did not affect [Cl^−^]_i_. In agreement with this lack of an effect of phasic GABAergic inputs on [Cl^−^]_i_, the compartmental simulations in NEURON revealed no major shifts in [Cl^−^]_i_ upon realistic background activation of synaptic GABAergic inputs. In addition, the simulations demonstrated that moderate enhancement of either frequency or conductance of GABAergic synaptic inputs induced only small changes in [Cl^−^]_i_. Only at frequencies > 10 Hz substantial [Cl^−^]_i_ changes were induced. This observation is in contrast to Cajal-Retzius cells, where already moderate, physiological levels of synaptic GABAergic activity (3.6 ± 0.6 Hz) mediate substantial [Cl^−^]_i_ alterations^19^, and to neurons from juvenile hippocampal slice cultures, where ongoing phasic activity is required to allow dynamic changes in E_GABA_^51^. However, in the present simulations [Cl^−^]_i_ was determined in the soma, to match the modelling results with the experimental design of the gramicidin perforated patch experiments. Within the dendritic compartment these levels of GABAergic synaptic activity will induce substantial GABAergic [Cl^−^]_i_ transients^17^.

In addition, the present study demonstrated that inhibition of both, phasic and tonic GABAergic currents with picrotoxin^13^ evoked a small, but significant [Cl^−^]_i_ increase. In line with this observation, the pharmacological induction of extrasynaptic GABAergic currents with THIP led to a substantial reduction in [Cl^−^]_i_. This observation could be replicated in the NEURON simulations by the addition of tonic Cl^−^ conductances. Our experiments suggest that the substantial tonic current found in immature hippocampal neurons^52^, contributes to steady state [Cl^−^]_i_. The dissimilar effects of phasic and tonic GABAergic currents directly reflect the different levels of charge transfer mediated by the short phasic responses (ca. 0.015 pC/s at 2.13 Hz and a g_GABA_ of 169 pS) in contrast to the persistent but small amplitude tonic GABAergic currents (ca. 0.46 pC/s at 8.75 nS/cm^2^)^43^.

A variety of studies in the adult brain have shown that excessive GABAergic stimulation can induce Cl^−^-fluxes that exceed the capacity of active Cl^−^ transport and consequently increase [Cl^−^]_i_ and alter GABAergic responses^15,16,18,25,40,51,53^. The low capacity of the NKCC1-mediated Cl^−^ uptake makes immature neurons particularly susceptible to such effects. On the other hand, spontaneous, highly correlated activity transients are typical for developing neuronal systems^54^. These correlated activity transients are characterized by the synchronous activation of glutamatergic and GABAergic activity^55,56^. The resulting massive GABAergic inputs may exceed the capacity of transmembrane Cl^−^ transport and induce substantial [Cl^−^]_i_ changes. The depolarizing glutamatergic inputs during correlated network activity can even augment the GABAergic Cl^−^ fluxes and thus aggravate the [Cl^−^]_i_ alterations^57^. Indeed it has been demonstrated that such physiological bursts of activity can led to substantial [Cl^−^]_i_ shifts in the spinal cord^33,58^, neocortex^19^, and immature hippocampus^17^. Whereas in the adult system such changes will decrease GABAergic inhibition and thus contribute to the establishment of hyperexcitable states and reduced pharmacological responsiveness^18^, in immature neurons the activity-dependent [Cl^−^]_i_ reduction will decrease the excitatory potential of GABA_A_ receptor-mediated responses. And since shunting inhibition remains constant^55,59^, this activity-dependent [Cl^−^]_i_ decrease will augment the inhibitory potential of GABAergic responses. Therefore, the low capacity of Cl^−^ export in immature neurons may be an adaptation to prevent hyper-excitability mediated by depolarizing GABA responses, as has been originally suggested by Ben-Ari^3^. In addition, at least for the immature spinal cord it has been demonstrated that such activity-dependent [Cl^−^]_i_ transients can determine the frequency of spontaneous activity transients by temporarily reducing the excitatory effect of GABA^58^. Thus it is tempting to speculate that recurrent alterations in [Cl^−^]_i_ may also contribute to slow oscillatory phenomena in other regions of the developing nervous system.

In summary, the results of our present study demonstrate that the capacity of Cl^−^ accumulation is limited in immature hippocampal CA3 pyramidal neurons. This finding, in combination with a quantification in immature cortical neurons^4^ and the observation of activity-related [Cl^−^]_i_ transients in other immature tissues^19,33^, suggests that an unstable [Cl^−^]_i_ homeostasis may be an innate feature of immature neurons.

## Methods

### Slice preparation

All experiments were conducted in accordance with EU directive 86/609/EEC for the use of animals in research and the NIH Guide for the Care and Use of Laboratory Animals, and were approved by the local ethical committee (Landesuntersuchungsanstalt RLP, Koblenz, Germany). All efforts were made to minimize the number of animals and their suffering. Wistar rat pups of P4-7 were obtained from the local breeding facility and were deeply anesthetized with enflurane (Ethrane, Abbot Laboratories, Wiesbaden, Germany). After decapitation, the brains were quickly removed and immersed for 2–3 min in ice-cold standard artificial cerebrospinal fluid (ACSF, composition see below). Coronal slices (400 µm thickness) including the hippocampus were cut on a vibratome (HR2, Sigmann Elektronik, Hüffenhardt, Germany). The slices were stored in an incubation chamber filled with oxygenated ACSF at room temperature before they were transferred to the recording chamber.

### Data acquisition and analysis

Gramicidin-perforated whole-cell patch-clamp recordings were performed as described previously^4,13^ at 31 ± 1 °C in a submerged-type recording chamber attached to the fixed stage of a microscope (BX51 WI, Olympus). Pyramidal neurons in stratum pyramidale of the CA3 region were identified by their location and morphological appearance in infrared differential interference contrast image. Patch-pipettes (5–12 MΩ) were pulled from borosilicate glass capillaries (2.0 mm outside, 1.16 mm inside diameter, Science Products, Hofheim, Germany) on a vertical puller (PP-830, Narishige) and filled with pipette solution containing 130 KCl, 1 CaCl_2_, 2 MgCl_2_, 11 EGTA, 10 HEPES, 2 Na2-ATP, 0.5 Na-GTP (pH adjusted to 7.4 with KOH and osmolarity to 306 mOsm with sucrose). For gramicidin-perforated patch-clamp recordings 10–50 µg/ml gramicidin D (Sigma, St Louis, MO, USA) was added from a stock solution (1–2 mg/ml in DMSO) on the day of experiment. Experiments were omitted from analysis, when an instantaneous or constant shift in the muscimol/glycine reversal potential towards positive values, determined by the high [Cl^−^] of the pipette solution, occurred, as they indicate insufficient perforated-patch conditions.

Signals were recorded with a discontinuous voltage-clamp/current-clamp amplifier (SEC05L, NPI, Tamm, Germany), low-pass filtered at 3 kHz and stored and analyzed using an ITC-1600 AD/DA board (HEKA) and TIDA software. Input resistance and capacitance were determined from a series of hyperpolarizing current steps. The apparent cell surface was estimated using a specific capacitance of 2 µF/cm^2^. Spontaneous postsynaptic currents (sPSCs) were detected and analysed from whole-cell patch-clamp recordings according to their amplitude and shape by appropriate settings using Minianalysis Software (Synaptosoft, Fort Lee, NJ).

The [Cl^−^]_i_ was determined from the reversal potentials of GABAergic and glycinergic currents recorded under voltage-clamp conditions using the Goldman–Hodgkin–Katz equation:

$$E_{GABA} = \frac{RT}{{ZF}}*\ln \left( {\frac{{P_{Cl} \left[ {Cl^{ - } } \right]_{e} + P_{HCO3} \left[ {HCO_{3}^{ - } } \right]_{e} }}{{P_{Cl} \left[ {Cl^{ - } } \right]_{i} + P_{HCO3} \left[ {HCO_{3}^{ - } } \right]_{i} }}} \right)$$

For the calculation of [Cl^−^]_i_ from E_GABA_ we used a [Cl^−^]_e_ of 133.5 mM, an extracellular HCO_3_^−^ concentration ([HCO_3_^−^]_e_) of 24 mM, a [HCO_3_^−^]_i_ of 14.1 mM, and published values for the HCO_3_^−^ permeability of GABA (0.18) or glycine (0.11) receptors^38^. GABAergic and glycinergic currents were evoked by brief (2–10 ms) pulses of 30 µM muscimol or 0.2–1 mM glycine from a patch pipette positioned close to the soma via a custom built pressure application system (Lee, Westbrook, CT) at a pressure of 0.5 bar. The use of glycine pulses was necessary to allow the determination of [Cl^−^]_i_ in the presence of gabazine or picrotoxin, which eliminate GABAergic currents^13^.

All values were given as median ± interquartile range, in the panels median ± interquartile range was used for time-dependent plots, while summarized results were shown as box and whisker plots (minimum, first quartile, median, third quartile, maximum). For statistical analysis of unpaired data Mann–Whitney U-tests and for paired data Wilcoxon signed-rank test were used (Systat 11, Point Richmond, CA). Significance was assigned at levels of 0.05 (*), 0.01 (**) and 0.001 (***).

### Morphological reconstruction

For morphological reconstruction, 18 CA3 pyramidal cells were filled with biocytin under whole-cell conditions. From this 18 stained neurons, two typical cells were used for quantitative somatodendritic reconstruction after visual inspection. For this purpose, 0.5–1% biocytin (Sigma, Taufkirchen, Germany) was added to the pipette solution. After filling of the cells, slices were fixed for at least 24 h in 4% paraformaldehyde. Subsequently they were rinsed and incubated 60 min with 0.5% H_2_O_2_ and 0.8% Triton-X100 to inhibit endogenous peroxidases. After overnight incubation with an avidin-coupled peroxidase (ABC kit, Vectorlabs, Burlingame, CA, USA), slices were pre-incubated in 0.5 mM diaminobenzidine and subsequently developed in diaminobenzidine and 0.015% H_2_O_2_. The slices were finally rinsed, dehydrated slowly through alcohol and propylenoxide, and embedded in Durcupan (Fluka, Buchs, Switzerland). Reconstruction and morphological analysis of the biocytin-labelled neurons were performed using the 60 × oil-immersion objective (NA 1.4) of a Nikon Eclipse 80i (Nikon, Germany) attached to a computer system (Neurolucida; MBF Bioscience Europe). Data was corrected for tissue shrinkage after importing to the NEURON environment. For this purpose we used the values suggested by Staiger et al.^60^ and expanded the x-/y-dimensions by 12.5% and the z-dimension by 50%.

### Compartmental modeling

The reconstructed CA3 pyramidal cell (see above) was imported into the NEURON simulation program (neuron.yale.edu). The following passive parameters were used: *R*_*a*_ (specific axial resistance) = 35.4 Ωcm; g_*pas*_ (passive specific membrane conductance) = 17.05 nS/cm^2^; E_pas_ = -74.05 mV, *C*_*m*_ (specific membrane capacitance) = 1 µF/cm^2^. In addition, a tonic leak Cl^−^ conductance:

$${\text{I}}_{{{\text{tonic}}}} = \, \left( {{1}{-}{\text{P}}_{{{\text{GABA}}}} } \right) \, \cdot {\text{ g}}_{{{\text{tonic}}}} \cdot \, \left( {{\text{V}}{-}{\text{E}}_{{{\text{Cl}}}} } \right) \, + {\text{ P}}_{{{\text{GABA}}}} \cdot {\text{ g}}_{{{\text{tonic}}}} \cdot \left( {{\text{V}}{-}{\text{E}}_{{{\text{HCO3}}}} } \right).$$

with a conductance g_tonic_ of 8.75 nS/cm^2^ was inserted. Implementing these parameters in the reconstructed morphology resulted in a resting membrane potential of − 70 mV and an input resistance of 306 MΩ.

Cl^−^ diffusion and uptake were calculated by standard compartmental diffusion modeling^40,44^. To simulate intracellular Cl^−^ dynamics, we adapted our previously published model^40^. Longitudinal Cl^−^ diffusion along dendrites was modeled as the exchange of Cl^−^ between adjacent compartments. For radial diffusion, the volume was discretized into a series of 4 concentric shells around a cylindrical core and Cl^−^ was allowed to flow between adjacent shells^61^. The free diffusion coefficient of Cl^−^ inside neurons (D_Cl_) was set to 2 µm^2^/ms^53^. To simulate Cl^−^ uptake, a pump mechanism for transmembrane Cl^−^ transport was included. Cl^−^transport was modeled as exponential recovery of [Cl^−^]_i_ to its target [Cl^−^]_i_ ([Cl^−^]_i_^0^) with a time constant τ_Cl_.

$$\frac{{{\text{d}}\left[ {{\text{Cl}}^{ - } } \right]_{{\text{i}}} }}{{{\text{dt}}}} = \frac{{\left[ {{\text{Cl}}^{ - } } \right]_{{\text{i}}}^{0} - {\text{e}}\left[ {{\text{Cl}}^{ - } } \right]_{{\text{i}}} }}{{{\uptau }_{{{\text{Cl}}}} }}$$

The pump mechanism approximates an NKCC1-like Cl^−^ transport mechanism.

The impact of GABAergic Cl^−^ currents on [Cl^−^]_i_ was calculated as:

$$\frac{{{\text{d}}\left[ {{\text{Cl}}^{ - } } \right]_{{\text{i}}} }}{{{\text{dt}}}} = \frac{1}{{\text{F}}}\frac{{{\text{I}}_{{{\text{Cl}}}} }}{{{\text{volume}}}}$$

with F = 96,485 C/mol (Faraday constant). GABA_A_ synapses were simulated as a postsynaptic parallel Cl^−^ and HCO_3_^−^ conductance with exponential rise and exponential decay^40^:

$${\text{I}}_{{{\text{GABA}}}} = {\text{ I}}_{{{\text{Cl}}}} + {\text{ I}}_{{{\text{HCO3}}}} = \, \left( {{1}{-}{\text{P}}} \right) \, \cdot {\text{ g}}_{{{\text{GABA}}}} \cdot \, \left( {{\text{V}}{-}{\text{E}}_{{{\text{Cl}}}} } \right) \, + {\text{ P }} \cdot {\text{ g}}_{{{\text{GABA}}}} \cdot \left( {{\text{V}}{-}{\text{E}}_{{{\text{HCO3}}}} } \right)$$

where P is a fractional ionic conductance that was used to split the GABA_A_ conductance (g_GABA_) into Cl^−^ and HCO_3_^−^ conductance. E_Cl_ and E_HCO3_ were calculated from Nernst equation. The GABA_A_ conductance was modeled using a two-term exponential function, using values of rise time (0.5 ms) and decay time (37 ms)^17^. Parameters used in our simulations were as follows: [Cl^−^]_o_ = 133.5 mM, [HCO3^−^]_i_ = 14.1 mM, [HCO3^−^]_o_ = 24 mM, temperature = 31 °C, P_GABA_ = 0.18, and P_Gly_ = 0.11^38^. The GABA_A_ inputs (107 synapses, peak conductance 0.169 nS)^17^ were activated stochastically (Poisson) with a frequency of 0.02 Hz, corresponding to a main PSC frequency of 2.14, except where noted. Source codes of all models are available at ModelDB (https://modeldb.yale.edu/266811; password is “hippocampus”).

### Solutions and drugs

The bathing solution consisted of 126 NaCl, 26 NaHCO_3_, 1.25 NaH_2_PO_5_, 1 MgCl_2_, 2 CaCl_2_, 2.5 KCl, 10 glucose (pH 7.4, osmolarity 306 mOsm) and was equilibrated with 95% O_2_ / 5% CO_2_ at least 1 h before use. GABA (γ-amino butyric acid), 6-Imino-3-(4-methoxyphenyl)-1(6H)-pyridazinebutanoic acid hydrobromide (gabazine, SR-95531), picrotoxin (PTX), 4,5,6,7-tetrahydroisoxazolo[5,4-c]-pyridin-3-ol (THIP), glycine and bumetanide were purchased from Sigma, and DL-2-Amino-5-phosphonopentanoic acid (APV), 6-Cyano-7-nitroquinoxaline-2,3-dione (CNQX), muscimol and tetrodotoxin (TTX), from Biotrend (Cologne, Germany). TTX and glycine were dissolved in distilled water and picrotoxin, gabazine, muscimol, bumetanide, CNQX and APV in dimethylsulfoxide (DMSO). All substances were added to the solutions shortly before the experiment. The DMSO concentration of the final solution never exceeded 0.2%.
